# Short-pulse laser plus subthreshold diffuse laser for serous retinal detachment in dome-shaped macula

**DOI:** 10.1186/s40942-023-00483-8

**Published:** 2023-09-01

**Authors:** Murilo Wendeborn Rodrigues, Thais Bastos, Annelise Nicotti Gonçalves, José Augusto Cardillo, André Messias, Eduardo Cunha de Souza, Rodrigo Jorge

**Affiliations:** 1 Division of Ophthalmology, Ribeirão Preto Medical School, University of São Paulo, Ribeirão Preto, SP Brazil; 2 Department of Ophthalmology, CRESEP Institution, Araraquara, Brazil; 3 Department of Ophthalmology, University of São Paulo, São Paulo, Brazil

**Keywords:** Short-pulse laser, Subthreshold diffuse laser, Dome-shaped macula

## Abstract

**Background:**

First described by Gaucher and associates in 2008, dome-shaped macula (DSM) is an anterior convex protrusion of the macula visible on OCT (optical coherence tomography). Visual impairment in DSM results mainly from sub-foveal serous retinal detachment (SRD). Herein, this original study from retrospective data analysis evaluate the anatomical and functional effects of Pascal^®^ short-pulse (SP) laser plus endpoint management (EpM) subthreshold diffuse laser (SDL) in patients with SRD due to DSM.

**Methods:**

This retrospective study included seven consecutive patients (eight eyes) with SRD secondary to dome-shaped macula who underwent a comprehensive ophthalmological evaluation including logMAR BCVA, slit-lamp biomicroscopy, indirect ophthalmoscopy, and spectral-domain optical coherence tomography (SD-OCT) (Spectralis; Heidelberg Engineering, Germany) before combined Pascal^®^ SP laser plus EpM-SDL with 1 to 6 month intervals, postoperatively, with a mean ± standard error (SE) follow-up time of 12.92 ± 1.34 months.

**Results:**

Eight eyes from seven patients were analyzed in this study. At baseline, mean BCVA (LogMAR) ± standard error (SE) and mean CST (central subfield thickness)(µm) ± SE were 0.6125 ± 0.14 and 412.50 ± 24.65, respectively. After a mean follow-up time of 12.92 ± 1.34 months, mean CST (µm) ± SE and BCVA (LogMAR) ± SE were 294.75 ± 19.68 (p = 0.0078) and 0.4537 ± 0.12 (p = 0.0313), respectively. A statistically significant reduction in mean CST and an improvement in mean BCVA were noted after SRD resolution with laser therapy application. The mean serous retinal detachment resolution time (months) ± SE was 3.75 ± 1.08. No adverse events were registered, including enlargement of atrophic alterations and choroidal neovascularization.

**Conclusions:**

The novel combined laser modality with Pascal^®^ SP laser plus EpM-SDL treatment may induce subretinal fluid regression and BCVA improvement 1 year after treatment in DSM patients with SRD.

## Introduction

Dome-shaped macula (DSM) was initially described by Gaucher in 2008 using optical coherence tomography (OCT) as an entity characterized by a convex elevation of the macula within a myopic staphyloma [1]. Serous retinal detachment (SRD) might be a possible complication of DSM, causing further deterioration of visual acuity [2]. Miscellaneous treatment options for SRD secondary to DSM [e.g., dorzolamide [3], spironolactone [4], eplerenone [5], micropulse laser [6], photodynamic therapy (PDT) [7], and intravitreal anti-VEGF [8]] have shown promise; however, inconstant results have been reported [9]. Thus, the aim of the present study was to describe the results obtained with combined sublethal laser techniques, namely, Pascal^®^ short-pulse (SP) laser plus EpM subthreshold diffuse laser (SDL), to treat SRD in DSM.

## Methods

### Study design

The present study was carried out in accordance with the Declaration of Helsinki and was approved by the Research Ethics Committee of the Ribeirão Preto Medical School of the University of São Paulo (Protocol No. 5.544.545). Data were collected in a retrospective fashion, and included the medical records of consecutive patients attended from September 2017 to January 2022 at a public vitreoretinal subspecialty practice who had undergone combined sublethal laser techniques (SP plus SDL therapy) to treat SRD secondary to DSM.

### Patient eligibility

The inclusion criteria were as follows: (1) 18 years old or above; (2) BCVA between 0.3 logMAR (Snellen equivalent: 20/32) and 1.3 logMAR (Snellen equivalent: 20/400); and (3) DSM associated with SRD by SD-OCT treated with combined laser modalities (Pascal SP laser plus EpM-SDL). Exclusion criteria: any evidence of vitreomacular traction by SD-OCT; no history of vitreoretinal surgery; no other ophthalmic disease other than cataracts and myopia.

### Patient demographics and evaluations

Patient data retrospectively assessed included: age, sex, lens status, and presence or absence of myopia/high myopia and/or pachychoroid (Table 1). All patients studied had been submitted to a comprehensive ophthalmic examination comprising a logMAR BCVA test with an ETDRS vision chart, slit-lamp biomicroscopy, indirect ophthalmoscopy, near-infrared reflectance (near-IR) scanning laser ophthalmoscopy, and spectral-domain optical coherence tomography (SD-OCT) (Spectralis; Heidelberg Engineering, Germany) through all visits from baseline until last visit period of analyzed data. The SD-OCT acquisitions included 9 mm horizontal and vertical scans centered on the fovea with enhanced depth imaging (EDI) analysis. Mean BCVA (logMAR) and mean CST (µm) were also evaluated and used as the main parameters for statistical analysis comparing baseline versus last follow-up values.

**Table 1:** Demographic population data and additional anatomical and visual ocular laser mean time information effects

Baseline characteristics		
Age mean (years) (± SD)		50.50 ± 2.72
Sex (female) (%)		100
Phakic (%)		100
Myopia (%)		100
High miopia (≥ 5D) (%)		50
Pachychoroid (%)		0
Follow-up laser therapy		
Mean time disease duration before laser therapy (years)(± SD)		2.31 ± 0.57
Laser follow-up mean time duration (month)(± SD)		12.92 ± 1.34
SRD mean time resolution (months)(± SD)		3.75 ± 1.08
Dried retina mean time follow-up (months)(± SD)		6.87 ± 1.37
BCVA mean change improvement (logMAR)(± SD)		0.159 ± 0.046
CST mean change decreased (μm)(± SD)		117.75 ± 22.7

### Treatment regimens

**Fig. 1:**
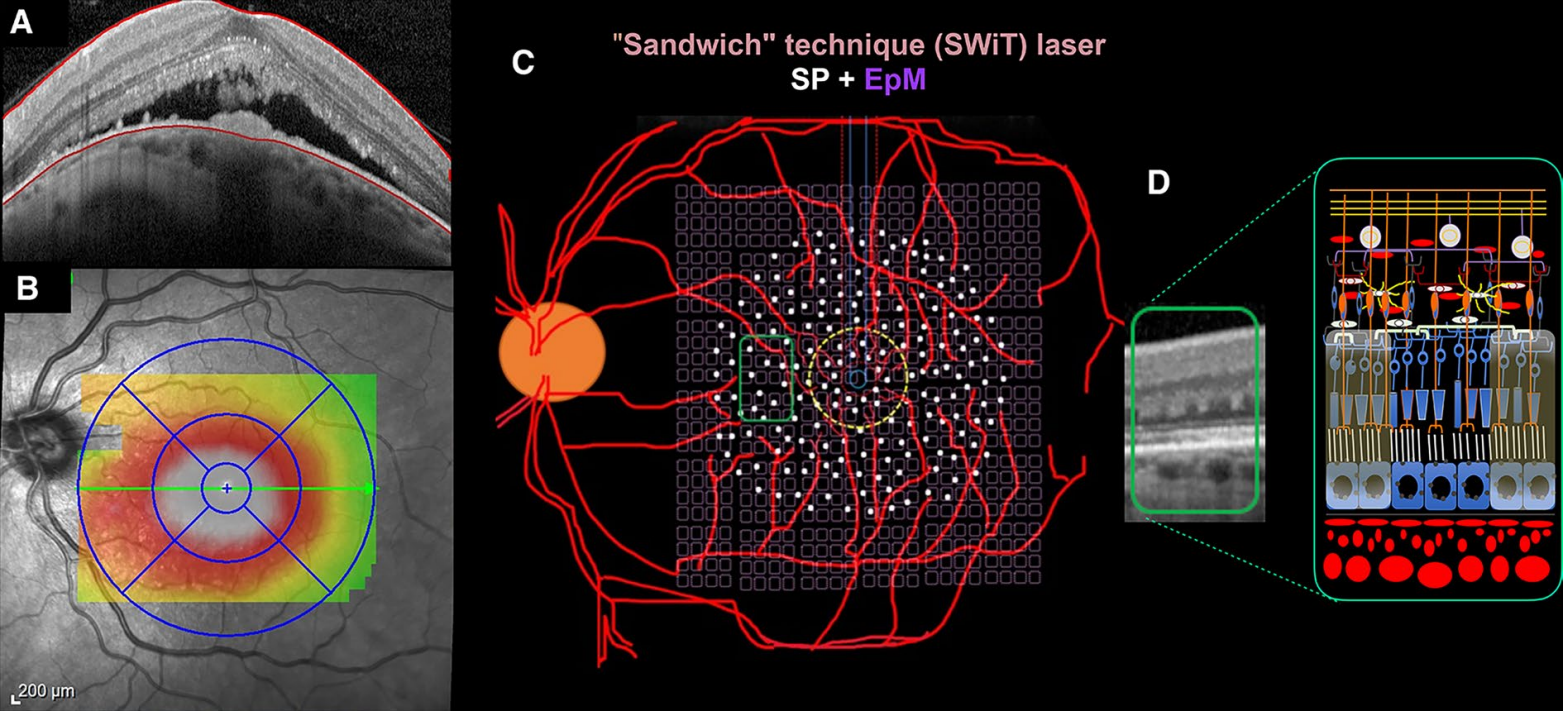
Configuration of the combined laser modalities. **A** SD-OCT in DSM case No. 6. **B** Thickness map with central, inner, and partial outer subfield foveal involvement. **C** Ocular fundus image illustrating both modalities from SWiT laser overlapped. **D** Green rectangle showing retinal parafoveal section with hyperreflective outer retinal laser sign after laser therapy

**Fig. 2:**
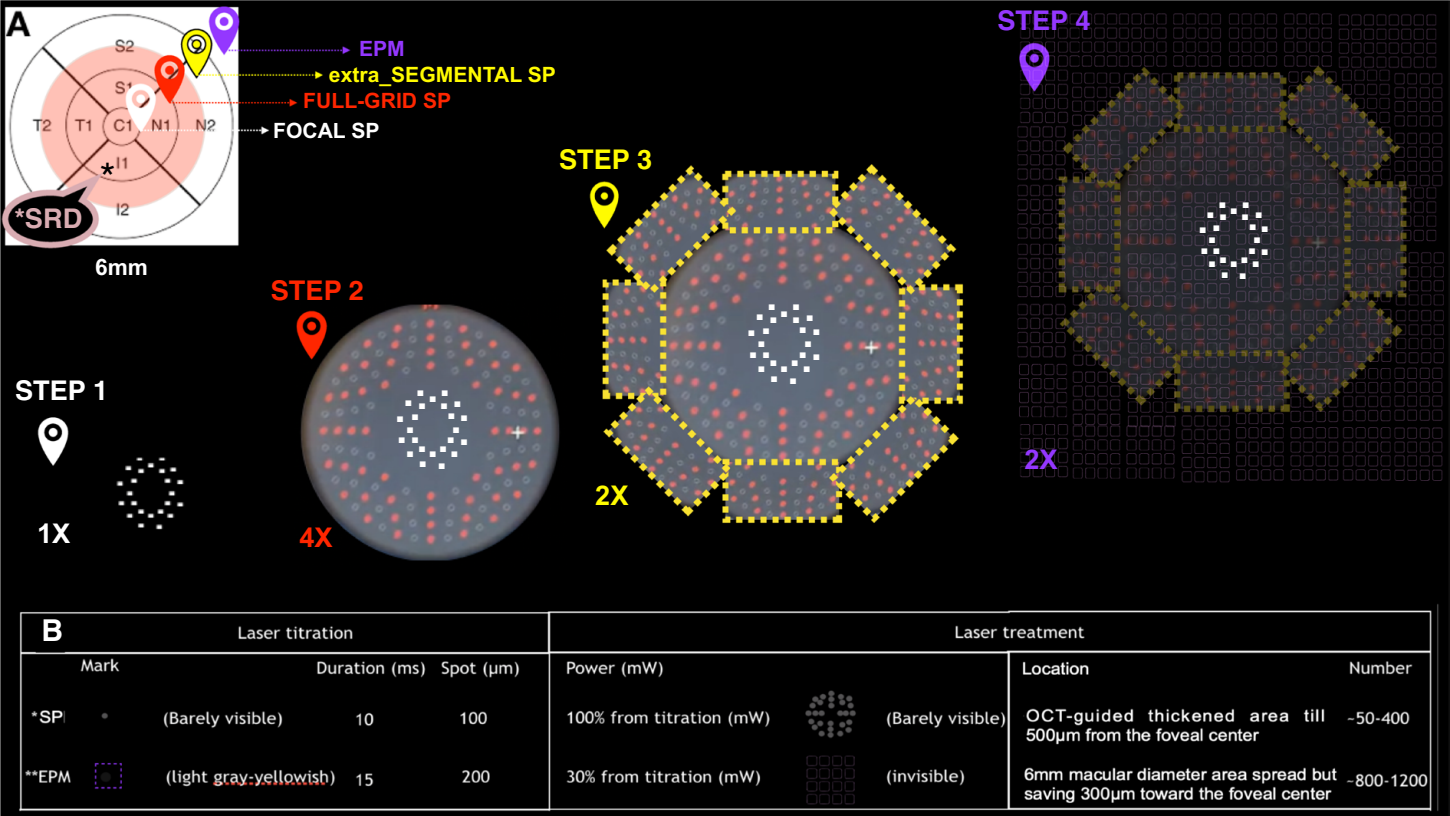
Step by step procedure execution of combined laser therapy and technique parameters. **A** Representative ETDRS map illustrating SD-OCT thickened zone (light pinkcolor) with SRD (*) involving CST, inner and partial outer subfield map. Step 1 (white): SP laser application single shots performing two or three circumferential arc precisely placed 360º around the foveal center, at a distance ranging from 500 µm far away from center foveola till 1000 µm. Step 2 (red): additional Pascal^®^ SP multiple full-grid spots were applied in a 1.100–1.600 radius from foveal center (Pascal^®^ octants grid pattern) which includes 56 spots simultaneously triggered on mode A (twice triggered) switched with 56 spots simultaneously triggered on mode B (twice triggered). Step 3 (yellow): eight extra-segmented 14 number spots of SP laser (twice trigged each) are performed outside the 1600-radius area, achieving a laser treated area that has approximately 1850 radius placed 360º around previous Pascal^®^ SP multiple full-grid spots marks. Step 4 (purple): EpM-SDL was applied sweeping (2 times) the posterior pole at a 3000 µm radial extension from the foveal center covering 6 mm by 6 mm square area, sparing the 300 µm distance from it and overlapping the SP laser-treated areas. **B** Technique parameters table: Pascal^®^ SP and EpM algorithm titration of each (power of mark of laser spot, laser duration and size of spot) and laser treatment mode (power, location and mean number of shots)

All treatments were provided using a Pascal^®^ laser unit, which uses a 532 nm frequency-doubled solid-state green laser source. An expert physician performed the combined laser therapy on all patients. The combined laser technique has been described recently for the treatment of DME cases and is known as “the sandwich technique” (SWiT) [10] (Fig. 1). Briefly, the combined sublethal laser therapy involved two photothermal stimulation methods: (1) sublethal SP laser (10 ms duration, 100 µm spot size, and barely visible mark from 100% power of titration, with a mean number of shots between 200 and 400) (Fig. 2). Once the titration power was obtained for barely visible spots placed in the extramacular area, the next step was to perform SP laser application individualy (Fig. 2A, step 1), with a few consecutive single shots performing two or three circumferential arcs precisely placed 360º around the foveal center, at a distance ranging from 500 µm from foveola center till 1000 µm. Afterward, additional Pascal^®^ SP multiple full-grid spots (Pascal^®^ octants grid pattern) were applied in 1100–1600 radius from foveal center (Fig. 2A, step 2) which includes 56 spots simultaneously triggered on mode A (twice triggered) switched with 56 spots simultaneously triggered on mode B (twice triggered). After, eight extra-segmented 14 number spots of SP laser (twice trigged each) are performed outside the 1600-radius area, achieving a laser treated area that has approximately 1850 radius (Fig. 2A, step 3) placed 360º around previous Pascal^®^ SP multiple full-grid spots marks. (2) EpM-SDL using the EpM algorithm software (approximately 30% titration power, 15 ms duration, and 200 µm spot size, with a mean number of shots between 800 and 1200) (Fig. 2A, step 4). Once again, titration power was set based on the generation of light gray barely visible burns placed in the extramacular area. The next step was applied using a 16-shot square grid pattern, with 0.25 mm spacing diameter apart, sweeping (2 times) the posterior pole at a 3000 µm radial extension from the foveal center covering a 6 mm by 6 mm square area, sparing the 300 µm radius from its center and overlapping the SP laser-treated areas.

### Statistical analysis

Data were collected in a retrospective manner through medical chart review. Statistical analysis was carried out using a single-arm univariate Student t-test, with statistical significance corresponding to a P value of 0.05 or lower using the JMP 10.0.0 software (2010; SAS Institute Inc, Cary, North Carolina, USA). Mean BCVA and CST measured after combined laser therapy were compared with the mean baseline values of the two parameters using multiple analysis of variance (MANOVA) for repeated measures.

## Results

**Fig. 3:**
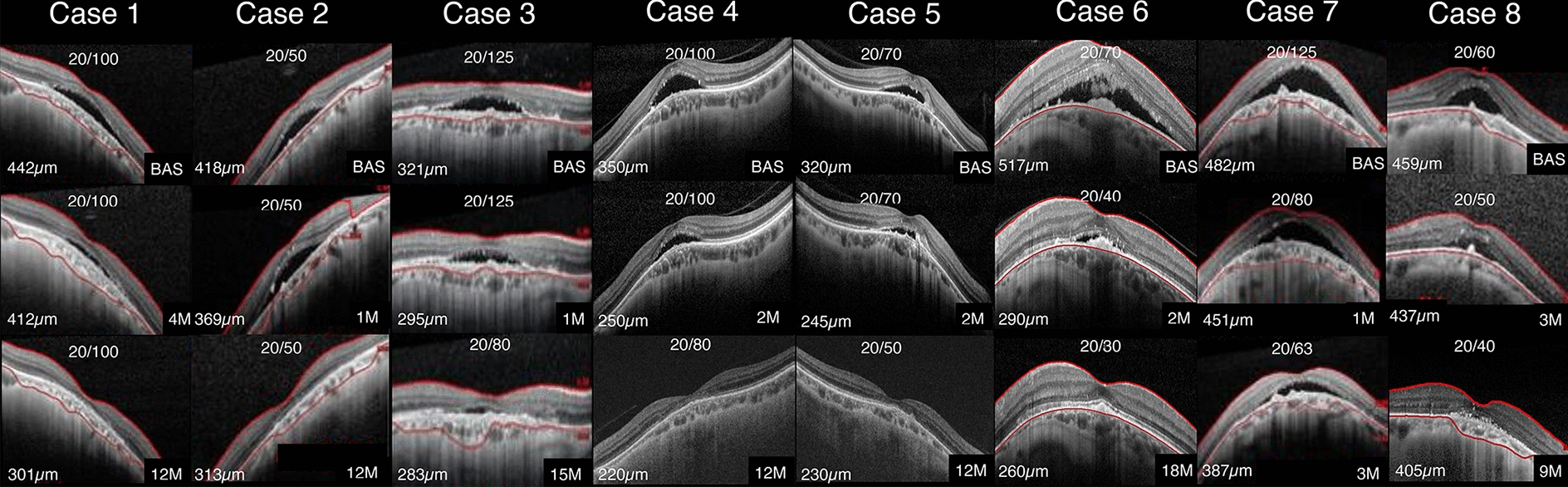
SD-OCT data follow-up of all cases from baseline to the last visit. SD-OCT of eight eyes showing seven cases of SRD resolution, with the exception of case No. 7

**Fig. 4:**
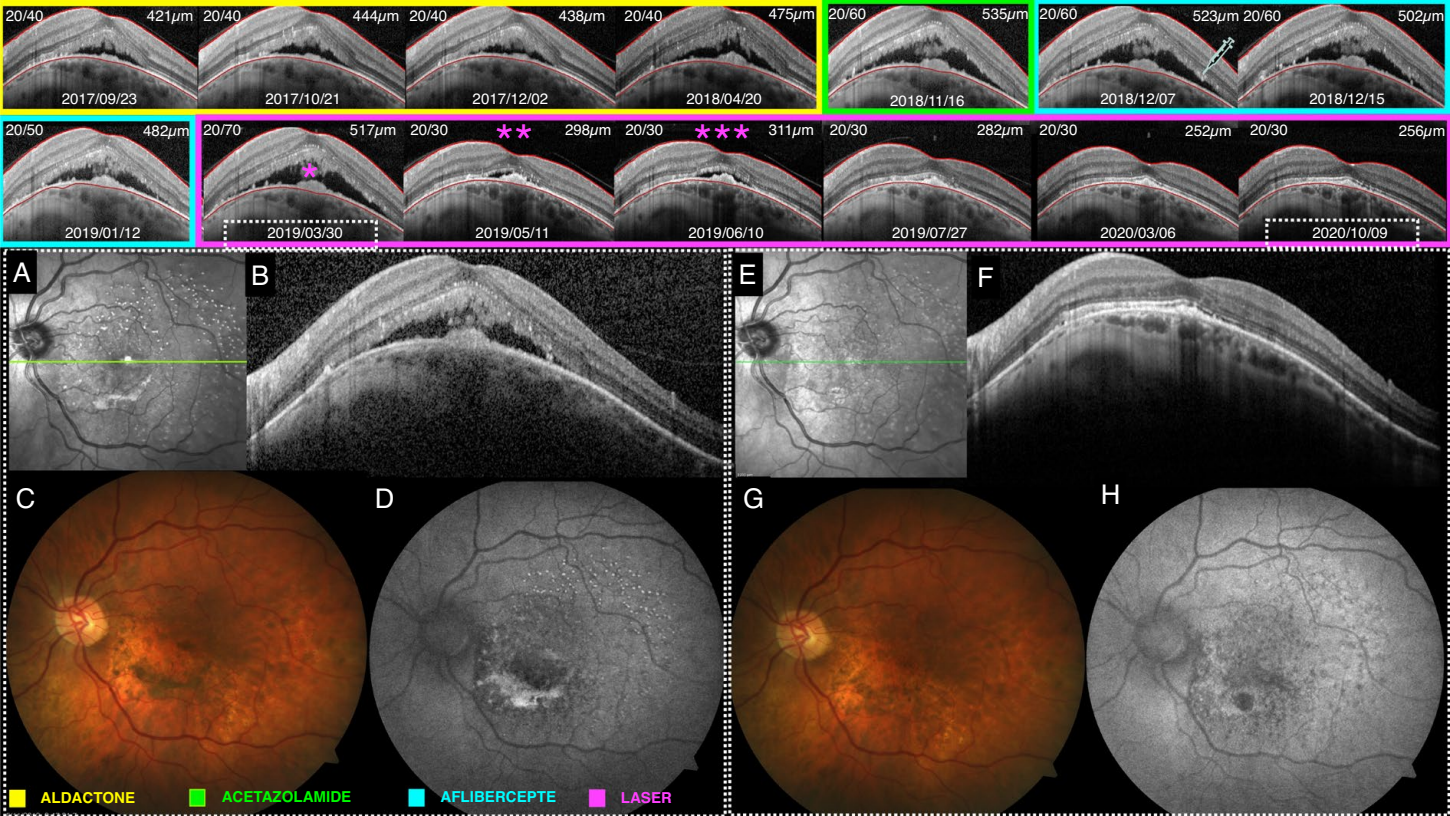
Patient who underwent several treatments for 2 years after symptom onset before combined laser therapy. (yellow) Fourteen months of SD-OCT follow-up with intermittent Aldactone^®^ treatment. (green) Acetazolamide daily therapy for 30 days of SD-OCT follow-up. (blue) Thirty days of SD-OCT follow-up after aflibercept intravitreous injection. (pink) Seventeen months of SD-OCT follow-up after combined laser therapy (*) with two additional treatments (**)(***), showing SRD resolution in four months. *Baseline multimodal imaging on the day of laser therapy (white dashed square)*: **A** Near-infrared imaging showing hypereflectance dots within para- and perifoveal areas. **B** SD-OCT illustrating the SRD in the DSM. **C** Color fundus retinography. **D** Autofluorescence showing hyperautofluorescent dots within para- and perifoveal areas and hyperautofluorescent amorphic material inferior to the macula. *Multimodal imaging on the last visit (white dashed square)*: **E** Near-infrared imaging showing faded hypereflectance dots within para- and perifoveal areas. **F** SD-OCT illustrating SRD resolution. **G** Color fundus retinography. **H** Autofluorescence showing hypoautofluorescent dots within para- and perifoveal areas and small hypoautofluorescent area from previous amorphic material located inferiorly to the macula

From September 2017 to January 2022, data from 8 eyes (mean age of 50.5 ± 2.7 years; 100% women) were enrolled in this study. Almost ninety percent (87.5%) of patients (7/8) had SRD resolution within 3.75 ± 1.08 months following laser treatment. One case (No. 7, Fig. 3), in which the patient had longstanding SRD in the past (> 2 years), presented a weak response regarding BCVA improvement and partial SRD resolution (Table 2, case No. 7). All cases mean follow-up period was 12.92 ± 1.34 months after laser therapy. The mean SRD resolution time was 6.87 ± 1.37 months. All patients had already received previous treatment, including eyedrops, oral medication, and scarce subthreshold micropulse laser applications. For example, case 6 (Fig. 3) had experimented several sorts of treatments before our combined laser therapy (Fig. 4). The laser therapy was allowed 2 months forward the last treatment failed. All cases had similar gap interval about 2 months before start laser procedure. Retreatment decision were allowed according the physician judgement analysis. Once the patient have received a first set of laser combined technique protocol, then it was evaluated monthly and additional laser approach was add till get satisfactory multimodal imaging fundus proving right accomplishment for the technique designed (i.e.: Fig 4, *asterisks in highlighted purple figures, had three sets of laser (1 month interval) before be considered adequately).

**Table 2:** Individually (8 eyes) anatomical and visual results before and after laser therapy

Cases	*CMT (μm)	**CMT (μm)	*BCVA (logMAR)	**BCVA (logMAR)
1	442	301	20/100 (0.69)	20/100 (0.69)
2	418	313	20/50 (0.39)	20/50 (0.59)
3	321	283	20/125 (0.79)	20/80 (0.60)
4	350	220	20/100 (0.69)	20/80 (0.60)
5	320	230	20/70 (0.54)	20/50 (0.39)
6	517	260	20/70 (0.54)	20/30 (0.17)
7	482	387	20/125 (0.79)	20/63 (0.49)
8	459	405	20/60 (0.47)	20/40 (0.30)
Mean	412.5	294.75	0.6125	0.4537
p	Baseline	p = 0.0078	Baseline	p = 0.0313

CMT (µm) column: (*) baseline and (**) forward laser therapy results. BCVA (logMAR) column (*) baseline and (**) forward laser therapy results

### Effect of treatment on retinal thickening

**Fig. 5:**
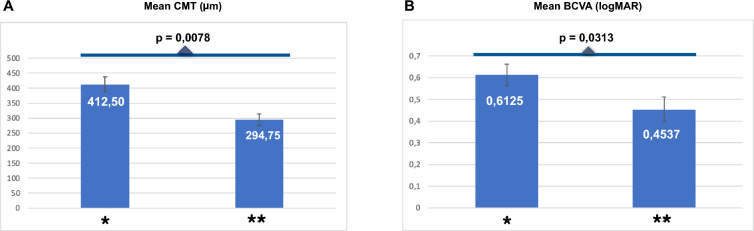
**Bar chart**: Mean anatomical and visual results before and after laser therapy. **A** mean central macular thickness (CMT) bar chart before (*) and after laser therapy (**), demonstrating statistically significant CMT reduction (p = 0,0078). **B** BCVA bar chart before (*) and after laser theraphy (**). Demonstrating statistically significant BCVA reduction (p = 0.0313)

The mean CST (µm) was 412.50 ± 24.65 at baseline and decreased significantly to 294.75 ± 19.68 after combined laser therapy (p = 0.0078). Five eyes (5/8, 62.5%) had a CST decrease of 100 µm or higher. The mean CST (µm) ± SE reduction at the last follow-up was 117.75 ± 22.7 (Table 2, Fig. 5A).

### Effect of treatment on visual acuity

The mean BCVA (LogMAR) was 0.6125 ± 0.14 (Snellen equivalent: 20/80) at baseline, which improved significantly to 0.4537 ± 0.12 (Snellen equivalent: 20/60) after combined laser therapy (p = 0.0313). Six eyes (6/8, 75%) showed BCVA improvements of 5 or more letters. The mean BCVA (LogMAR) ± SE improvement after laser treatment was approximately 0.159 ± 0.046 (Table 2, Fig. 5B).

### Adverse effects

None of the patients complained about increased central scotoma. No laser scars were verified on the central fovea. No heavy burns occured in the macular area treated with the combined laser technique.

## Discussion

Retrospective reports with small data analysis and short-term follow-up for DSM have described several treatment methods for SRD, with inconsistent results, a fact that hampers precise conclusions as to the best therapeutic approach (example in Fig. 4, case No. 6 underwent several treatments before laser therapy) [9]. Thus, our group decided to attempt a combined laser technique published in the study by Cardillo et al. [10], called “the sandwich technique” (SWiT), which showed favorable anatomical and visual outcomes for DME treatment.

SWiT laser therapy uses a sublethal low-intensity threshold laser (i.e., SP laser) and a subthreshold invisible diffuse laser modality (i.e., EpM-SDL). The former produces transient low and focal thermal elevations in the retinal pigment epithelium (RPE) and, consequently, SD-OCT hyperreflective signs in the outer retinal layer (Fig. 1D) with very little lateral spread of heat from the RPE, leaving the inner retina intact [11]. In theory, clinical response could be related to laser-induced activation of the heat shock protein (HSP-70), triggering a subsequent initiation step in a cascade of reparative phenomena that improve RPE function [12]. Over time, these lesions tend to contract instead of expand, and healing takes place after a few months, providing the reestablishment of the photoreceptor layer and local synapses between migrated photoreceptors and preserved bipolar cells [13] (Fig. 4A and E). Unlike PDT (large spot size targeting the entire foveal area), these visible focal SP laser marks can be applied safely with the advent of a Pascal^®^ multi-spot device, avoiding the risk of retracting the same macular areas and respecting the 500µm distance from the foveal center [14, 15] (Fig. 1C, 2A step 1–3).

The additional SDL technique (EpM algorithm) allows for high-density shot overload and the safe treatment of juxtafoveal areas up to 300 µm from the foveal center. High-density SDL must be delivered twice to the entire posterior pole (3 mm radius from the foveal center), thus maximizing therapeutic recruitment of RPE cells and activating microglial cells [16–21] (Fig. 1C, 2A step 4).

In our study, disease mean duration reported before the combined laser therapy modality was approximately 2.31 ± 0.57 years (Table 1), and, despite the chronicity of SRD preceding our laser treatment, there was mean BCVA and CST improvement. A total of seven cases (7/8, 87.5%) exhibited full SRD resolution in a mean time of around 3.75 ± 1.08 months following combined laser therapy. The total mean follow-up duration after laser therapy was 12.92 ± 1.34 months (Table 1), and the patients who presented SRD resolution maintained stable anatomical and visual results until the last visit (Fig. 3). Only one case (No. 7, Fig. 3) showed partial SRD absorption, with little visual acuity improvement after the combined laser therapy approach. The previous long-term SRD duration (more than 2 years) and consequent external retinal layer/RPE impairment (i.e., subretinal pigment clumps, flat irregular pigmented epithelium detachment, macular RPE atrophy, and ellipsoid band irregularities) may justify the limited anatomical results reported previously [22].

Soudier et al. [22] published results on the long-term evolution of DSM in 29 eyes over 37.89 ± 33.04 months of mean follow-up. Approximately 51% (15/29) of the patients presented with SRD at baseline, while three others (10%) developed SRD during follow-up. Despite the several therapeutic strategies offered to 12 eyes (including anti-VEGF injections, half-fluence PDT, triamcinolone acetonide, focal laser, and oral eplerenone), none of the SRDs regressed or resolved during the treatment period or immediately thereafter. Complete absence of SRD was observed in 11 eyes (38%) during the entire follow-up period; however, the visual outcomes did not change after treatment in any of the cases. In the present study, we observed earlier SRD resolution (3.75 ± 1.08 months after combined laser therapy) and a higher rate of complete SRD resolution (87.5%) associated with significant visual improvement.

In addition, Soudier et al. [22] noticed that the size of macular RPE atrophy increases as the DSM bulge increases and that the presence of SRD is more likely to occur when the height of the bulge is greater than 350 µm. In our study, the measurements of bulge height and the size of RPE atrophy or SRD progression were not statistically analyzed. Moreover, our study had a shorter follow-up duration (mean of 12.92 ± 1.34 months) than the study mentioned above [22] (mean of 37.89 ± 33.04 months). The shorter follow-up in our study may have favored the lower recurrence rate of SRD.

Our study results corroborate those reported by Caillaux et al. [2], who found that the leading causes of visual impairment were related to SRD; however, the macular bulge height was not associated with poorer BCVA. The BCVA was significantly lower in eyes with SRD than in eyes without the condition (p = 0.043). Also, they observed that macular bulge height was positively correlated with choroidal thickness and the presence of foveal SRD, agreeing with the Soudier et al. [22] results mentioned above. In the present study, the relationship between bulge height and choroidal thickness was not statistically analyzed.

Pirani et al. [23] also used combined laser treatment strategies with half-fluence and half-dose photodynamic therapy (PDT) plus subthreshold 577 nm micropulse laser therapy (STLT) for SRD in DSM patients with the aim of targeting both choroidal and RPE dysfunction. Poorer results were reported compared to our study, with 45.4% and 54.5% of cases achieving total SRD resolution and two lines of improvement in the BCVA at the end of the 6 month follow-up, respectively. Meanwhile, our study yielded 87.5% and 75% for the same parameters, respectively.

The present study had some design limitations as a retrospective study, including a relatively small sample size and the lack of retinal sensitivity measurements such as microperimetry. More extensive prospective studies could provide further valuable insight into the combined laser modalities approach.

## Conclusion

Trials using sophisticated techniques are still scant in the ophthalmic literature. Expensive laser equipment, extensive physician training, and time-consuming procedures are factors that may discourage most specialists. In essence, our study shows that combined SPD and SDL techniques (first-time used in DSM disease) may be effective for SRD resolution and subsequent visual function improvement in DSM patients.

## Data Availability

The datasets used and/or analysed during the current study available from the corresponding author on reasonable request.

## Abbreviations

DSM: Dome-shaped macula
SRD: Serous retinal detachment
SD-OCT: Spectral domain optical coherence tomography
SP: Short-pulse
EpM: Endpoint management
SDL: Subthreshold diffuse laser
BCVA: Best correct visual acuity
SE: Standard error
CST: Central subfield thickness
STLT: Subthreshold laser therapy
PDT: Photodynamic therapy
VEGF: Vascular endothelial growth factor
RPE: Retinal pigment endothelium
ETDRS: Early treatment diabetic retinopathy study
SWiT: Sandwich technique
